# Role of the Systemic Immune-Inflammation Index in Patients with Metastatic Renal Cell Carcinoma Treated with First-Line Ipilimumab plus Nivolumab

**DOI:** 10.3390/cancers14122972

**Published:** 2022-06-16

**Authors:** Viktoria Stühler, Lisa Herrmann, Steffen Rausch, Arnulf Stenzl, Jens Bedke

**Affiliations:** Department of Urology, University Hospital Tuebingen, 72076 Tuebingen, Germany; viktoria.stuehler@med.uni-tuebingen.de (V.S.); lisa.herrmann@student.uni-tuebingen.de (L.H.); steffen.rausch@med.uni-tuebingen.de (S.R.); arnulf.stenzl@med.uni-tuebingen.de (A.S.)

**Keywords:** immune checkpoint inhibitors, immuno-oncology, ipilimumab, nivolumab, renal cell carcinoma, systemic immune-inflammation index

## Abstract

**Simple Summary:**

The aim of this study was to evaluate the predictive and prognostic value of the systemic immune-inflammation index (SII), which is based on peripheral blood platelet, neutrophil, and lymphocyte counts, in patients with metastatic renal cell carcinoma (mRCC) treated with ipilimumab plus nivolumab in the first-line setting. High SII score was an independent prognostic factor for worse progression-free survival and overall survival. The clinical benefit rate was higher for patients with a low SII index if compared to a high SII index. An increase in SII of >20% from baseline after 12 weeks of therapy was significantly associated with tumor progression at first imaging. The SII index is both prognostic and predictive and could refine decision making in patients treated with ipilimumab plus nivolumab.

**Abstract:**

Background: The aim of this study was to evaluate the predictive and prognostic value of the systemic immune-inflammation index (SII) in patients with metastatic renal cell carcinoma (mRCC) treated with first-line ipilimumab plus nivolumab. Methods: This retrospective study included forty-nine mRCC patients treated with first-line ipilimumab plus nivolumab at the Department of Urology of the University of Tuebingen, Germany. SII was assessed before starting ipilimumab plus nivolumab therapy at the time of first imaging and at tumor progression. Optimal SII cut-off was stratified by ROC-analysis. Univariable and multivariable Cox regression analyses were used to evaluate the predictive and prognostic value of SII. Results: Optimal SII cut-off was 788. Twenty-nine/forty-nine patients had high SII (≥788) before initiation of ipilimumab plus nivolumab. High SII was an independent prognostic factor for worse progression-free (HR 2.70, *p* = 0.014) and overall survival (HR 10.53, *p* = 0.025). The clinical benefit rate was higher for patients with low SII if compared to high SII (80% vs. 32.1%). An increase in SII > 20% from baseline after twelve weeks of therapy was associated with progression at first imaging (*p* = 0.003). Conclusions: SII is both prognostic and predictive and could refine decision making in patients with unclear imaging on therapy with ipilimumab plus nivolumab.

## 1. Introduction

Approximately 25% of patients are diagnosed with renal cell carcinoma (RCC) at advanced stages or after metastasis (mRCC) [1]. In recent years, treatment options have expanded dramatically. Long-used tyrosine kinase inhibitor (TKI) monotherapies such as sunitinib, pazopanib, and cabozantinib have been almost completely replaced by immune checkpoint inhibitor (IO)-based therapies consisting of IO-IO (ipilimumab plus nivolumab) or TKI-IO combinations (axitinib plus pembrolizumab, lenvatinib plus pembrolizumab, or a cabozantinib plus nivolumab) [2,3]. With the addition of IO-based combination therapies, patient prognosis has improved dramatically. A meta-analysis of randomized clinical trials from 2021 showed a 26% decreased risk of death for IO combinations compared to sunitinib with a PFS benefit and higher complete response and overall response rates [4].

However, it has not yet been conclusively determined which combination therapy should be chosen in terms of an individualized approach. To date, the International Metastatic Renal Cell Carcinoma Database Consortium (IMDC) risk criteria are the gold standard for predicting the survival of mRCC patients and are therefore used in clinical practice for risk grouping. In addition to clinical findings such as Karnofsky performance status and the time interval from first diagnosis to the initiation of drug therapy, the IMDC variables also incorporates laboratory parameters such as hemoglobin, calcium, absolute neutrophil, and platelet count [5,6]. Therefore, an Eastern Cooperative Oncology Group performance status (ECOG) 0 and 1, patient age <65 years, and male sex reduced the risk of death with IO-TKI combination therapies [7].

The immune system, including the inflammatory response and tumor microenvironment, plays an important role in clinical and biological behavior as well as the outcome of RCC [8]. Inflammation-based prognostic scores such as platelet to lymphocyte ratio [9], lymphocyte to monocyte ratio [10], and prognostic nutritional index [11] are used in cancer patients [12]. The systemic immune-inflammation index (SII) combines three immune cell counts, neutrophils, lymphocytes, and platelets, into a simple formula and comprehensively maps the cancer-related inflammatory burden [13]. SII has shown a significant association with oncological outcomes in several malignancies [14,15], including urothelial cancer [16,17], pancreatic cancer [18], non-small lung and laryngeal cancer [19,20], and cholangiocarcinoma [21]. Studies investigating the prognostic value of SII in RCC patients reached controversial results. A meta-analysis for mRCC, which included ten studies with a total of 3.180 patients, showed that high SII was associated with poor OS (HR 1.75, 95% CI 1.33–2.30, *p* < 0.001). Studies included in this meta-analysis comprised non-metastatic RCC patients or patients treated with TKIs in the metastatic setting. Information for dual immune checkpoint inhibition with ipilimumab and nivolumab is missing [22].

We hypothesized that the SII score classifies mRCC patients according to their individual risk of progression on dual IO-IO combination therapy with ipilimumab plus nivolumab. In addition, we investigated the predictive and prognostic value of the SII index in patients with mRCC treated with ipilimumab plus nivolumab in the first-line setting.

## 2. Materials and Methods

Our study included 49 consecutive mRCC patients treated with first-line ipilimumab plus nivolumab at the Department of Urology of the University of Tuebingen, Tuebingen, Germany, as of May 2018. There were no specific exclusion criteria. Local research ethics committee approval was taken (078/2012/B02).

Input data for the SII score was obtained as follows: absolute number of neutrophils multiplied by absolute number of platelets divided by absolute number of lymphocytes. The SII score was assessed before starting the IO-IO combination, after 12 weeks at the time of first imaging, and at tumor progression.

The cohort was stratified using optimal SII cut-offs, first for initial analysis a cut-off of 854 was used as determined by using meta-analysis [22], second an optimal SII cut-off value was defined by generating a time-dependent receiver operating characteristic (ROC) curve to determine the Youden index. Based on this result, the total population was divided into two separate SII groups (SII high ≥788 vs. SII low <788). In addition to the SII score, clinical, pathological and treatment-related parameters were also recorded. Data collected included time of initial surgery, TNM stage, grading, histologic subtype, and Karnofsky performance status. The Memorial Sloan-Kettering Cancer Center (MSKCC) and the IDMC risk score at the time of first metastasis was calculated.

Categorical variables were tested using chi-squared tests or either Fisher’s exact test or Pearson’s chi-square test, whereas differences in continuous variables were analyzed with Mann-Whitney U tests as appropriate. PFS was defined as time from start of IO-IO treatment until disease progression or death. OS was defined as the interval between start of ipilimumab plus nivolumab until death from any cause or censored at the time of last follow-up. PFS and OS were analyzed using descriptive statistics and Kaplan-Meier curves. Univariable and multivariable Cox regression analyses were conducted to analyze the association of SII with PFS and OS. Statistical analyses were performed using SPSS, version 27. A *p* < 0.05 was considered statistically significant.

## 3. Results

### 3.1. Patient Characteristics

A total of forty-nine patients with a median age of 64.6 years (range 39.9–83.5 years) were included in this analysis; thirty-five (71.4%) were male. The histologic subtype was clear cell RCC in thirty-nine (79.6%) cases. Thirty patients (61.2%) presented with synchronous metastasis (Table 1).

Based on the meta-analysis by Jin et al., the calculated mean cut-off value for SII was 854 with a range of 529–1375 [22]. ROC analysis showed the optimal cut-off value to be 788 (sensitivity 82.6%, specificity 36.0%) for our collective. Of the forty-nine patients included in this study, twenty-nine patients (59.2%) were categorized into the high (SII ≥788) and twenty (40.8%) into the low SII (<788) group. The association between SII and baseline clinicopathological characteristics is shown in Table 1. There were no significant differences in clinicopathological parameters between patients with low and high SII scores (Mann-Whitney U test), including median time from primary tumor to metastasis as well as to initiation of therapy with ipilimumab plus nivolumab (*p* = 0.799 and *p* = 0.669, respectively). However, median duration of treatment with ipilimumab plus nivolumab was almost twice as long in the SII low group (6.64 vs. 3.42 months, *p* = 0.014). Responses were assessed by serious axial imaging, usually at twelve-week -intervals, and investigator assessments.

The median follow-up since initiation of ipilimumab plus nivolumab was 9.53 months (range 0.33–45.9 months) showing significant difference between groups with 17.4 months in the SII low group compared to 7.36 months in the SII high group (*p* = 0.008).

### 3.2. Survival Analysis

Median PFS for the overall population was 5.29 months (range 0.33–33.6 months). The 6- and 12-month PFS rates were 49.3% and 34.6%, with 73.7% and 45.9% for the SII low group and 31.3% and 27.4% for the SII high group.

**Figure 1:**
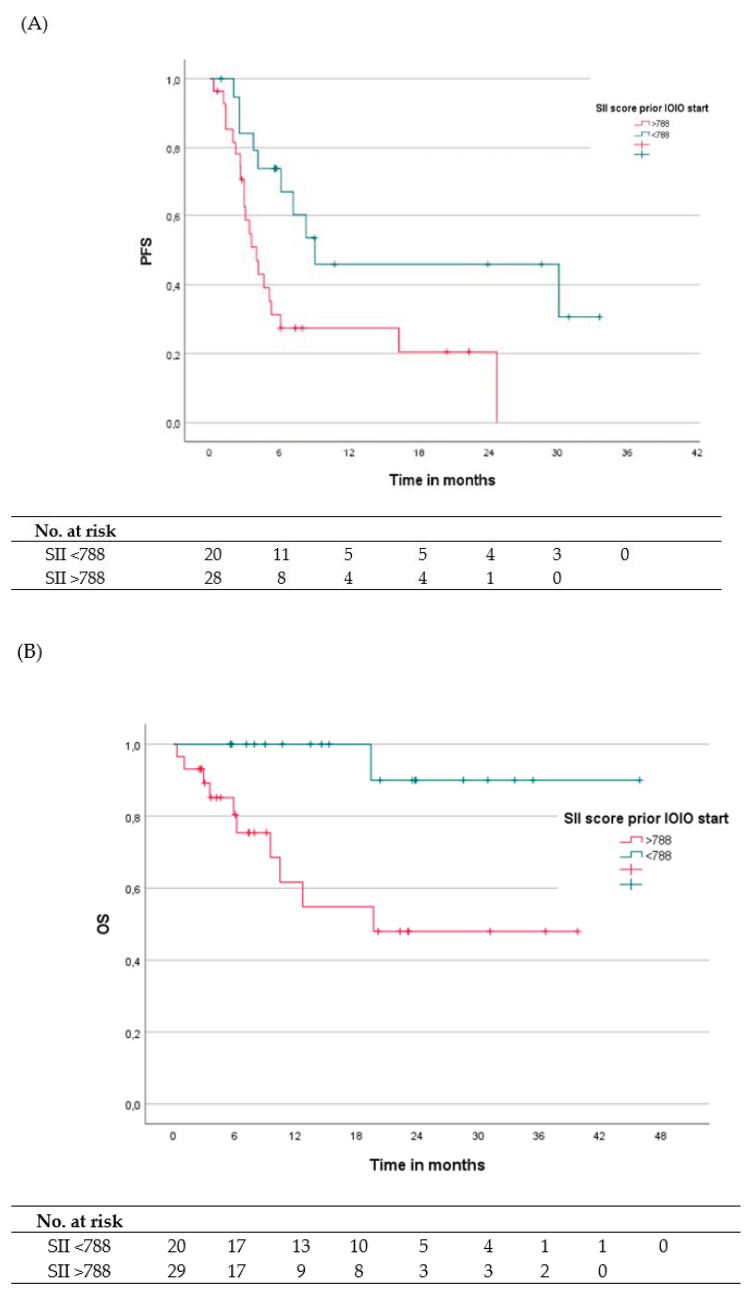
(**A**) Kaplan-Meier analyses illustrating PFS depending on SII score with a cutoff value of 788. PFS defined as time from start ipilimumab plus nivolumab to tumor progression. (**B**) Kaplan-Meier analyses for OS depending on SII score with a cutoff value of 788. OS is defined as time from start ipilimumab plus nivolumab to death/last follow-up. The 1-year PFS rates were 45.9% and 27.4% for SII <788 and ≥788, respectively, and the 1-years OS rates were 100% and 61.7% for SII <788 and ≥788.

Median OS for the overall population was not reached (range 0.33–45.9 months), with overall OS rates of 88.9%, 79.3%, 75.7%, and 67.3% at 6, 12, 18, and 24 months, respectively. OS rates were 100% at 18 months and 90% at 24 months in the low SII group and 80.4%, 61.7%, 54.8%, and 48% at 6, 12, 18, and 24 months, respectively, for the high SII group. The corresponding Kaplan-Meier curves are shown in Figure 1A,B.

A high SII score before starting ipilimumab plus nivolumab was an independent prognostic factor for worse PFS (4.01 vs. 9.04 months, HR 2.70, 95% CI 0.22–5.97, *p* = 0.014) and OS (19.60 months vs. NR, HR 10.53, 95% CI 1.34–82.68, *p* = 0.025). Other known prognostic factors examined, such as synchronous metastasis, time interval from RCC diagnosis to metastasis <1 year, intermediate/poor IMDC or MSKCC risk group, or baseline metastasis in multiple organ systems, did not significantly affect PFS and OS (see Table 2A,B). In multivariable analyses this prognostic effect of SII score could only be confirmed for PFS with an HR of 3.63 (*p* = 0.010), but not for OS with an HR of 6.91 (*p* = 0.068), see Table 3A,B.

Significant associations for SII with PFS and OS were also observed for the literature- based SII cut-off of 854 (*p* = 0.046 and *p* = 0.028, respectively). However, at the threshold of 788 calculated for this cohort, the correlations were even more pronounced, see Table 2A,B.

### 3.3. Response

In the overall population, clinical benefit rate (CBR) at first imaging was observed in twenty-five of forty-eight (52.1%) evaluable patients. Eighteen patients achieved partial response and seven patients showed stable disease, while twenty-three patients had progressive disease. The CBR at first imaging was 80% in patients with low SII, with twelve patients showing a partial response and four patients stable disease, while the CBR in patients with high SII was 32.1% with a documented partial response in six patients and stable disease in three patients. In the SII high group, nineteen of twenty-eight (67.9%) assessable patients showed progressive disease at first imaging.

Additionally, an increase in SII of >20% above baseline after twelve weeks on therapy was significantly associated with tumor progression at initial imaging (chi-square test *p* = 0.003, Table 3C).

During the observation period, a total of thirty-one patients showed tumor progression after a median of 3.58 months (range 1.18–30.12 months). At the time of documented progression, sixteen patients (51.6%) had an increase in SII > 20%, eight patients (25.8%) had stable SII, and seven patients (22.6%) had a decrease in SII > 20%.

## 4. Discussion

The efficacy of immune checkpoint inhibitors in mRCC varies widely between patients. Treatment decision is hampered by the lack of prognostic and predictive biomarkers that capture the inherent biological aggressiveness of the tumor as well as the host response and could help to overcome current staging and prognostic problems.

The immune system plays a critical role in tumor development via several mechanisms [23]. Inflammatory states extrinsically promote malignant cell proliferation and survival, angiogenesis, and metastasis, whereas activation of oncogenes drives intrinsic inflammatory pathways [24]. All immune cells that are assessed by the SII score play a pivotal role in the cancer response and cancer-related inflammation [24,25,26]. An impairment of immune cells by the developing tumor microenvironment can be detected in cancer patients [27].

Neutrophils in the tumor microenvironment can release various cytokines and chemokines including reactive oxygen species and transforming growth factor B to educate themselves and other cell types to differentiate into a cancer-promoting phenotype [28,29]. Further, tumor-associated neutrophils are highly responsible for modulating the tumor microenvironment, and increased neutrophil numbers may be primarily associated with treatment resistance [28]. Moreover, neutrophils interact with circulating tumor cells and facilitate binding to the endothelium. This interaction may promote tumor progression and metastasis by inducing tumor cell proliferation, stimulating angiogenesis, and suppressing adaptive immune response function in the tumor microenvironment [30,31].

Platelets are thought to protect tumor cells from elimination by the immune system and accelerate transendothelial migration and metastasis. Several platelet-secreted growth factors, including vascular endothelial tumor growth factor, platelet-activating factor, and platelet-derived growth factor, affect tumor growth and metastasis [9,32,33,34].

Cytotoxic lymphocytes play an important role in cell-mediated immunological killing of tumor cells [35]. Reduced lymphocyte numbers may be a consequence of tumor inhibition and have been associated with an impaired response to carcinogenesis [36].

Consequently, SII score is a comprehensive biomarker of inflammatory burden in mRCC and can be considered as an indicator of the balance between tumor and antitumor activities of the host immune response. Thus, a high SII resulting from an increased neutrophil or platelet count and/or a low lymphocyte count, can correlate with poor survival in tumor patients.

Recently, it has been reported that the SII sore is of prognostic value in many malignant tumor types including hepatocellular carcinoma [37], gastric cancer [38], colorectal cancer [39], and urological and gynecological tumors [13,40]. Given that RCC is an inflammatory disease, the investigation of the SII score seems to be of particular interest in patients treated with immune checkpoint inhibitors. Therefore, we retrospectively investigated the clinical value of the blood-based SII score in mRCC patients before starting first-line therapy with ipilimumab plus nivolumab. We found that patients with high SII were at significantly increased risk of shorter PFS and OS with first-line ipilimumab plus nivolumab. Our results suggest that a high SII may serve as a clinical guide to predict tumor aggressiveness with shorter PFS and OS. Conversely, a lower SII score prior to initiation of ipilimumab plus nivolumab appears to identify patients who may benefit from this combined therapy approach instead of a TKI-IO based combination.

Not only the level of the SII score before treatment but also its change during ipilimumab and nivolumab therapy was of prognostic value. Thus, an increase in SII of more than >20% from baseline after 12 weeks of therapy was significantly associated with tumor progression at initial imaging. This observation may help to distinguish between pseudo-progression or true progression on imaging as well as in cases of documented initial mixed response to immune checkpoint inhibitor-based therapy.

Data of a recent meta-analysis is in line with our data: A high SII was associated with poor OS (HR 1.75, 95% CI 1.33–2.30, *p < 0*.001) in an all-comer cohort of non-metastatic and metastatic RCC. Contrary to our data, high SII did not prove to be a significant prognostic factor for PFS in this meta-analysis (HR 1.22, 95% CI 0.84–1.76, *p* = 0.293) [22], which included TKI-treated patients. A study by Hu et al. included in this meta-analysis with non-metastatic RCC patients treated with nephrectomy found that high SII (>529) was an independent predictor of cancer-specific survival (HR 2.17, 95% CI 1.33–3.55, *p* = 0.002) and OS (HR 2.26, 95% CI 1.44–3.54, *p* < 0.001) [41]. This meta-analysis also included studies with mRCC patients treated with TKIs. For example, Basal et al. showed significant differences in OS between SII low (<730) and SII high groups (>730; 27.0 vs. 12.0 months, *p < 0*.001) in one hundred and eighty seven mRCC patients receiving first-line TKI therapy [42]. Lolli et al. included three hundred and thirty five consecutive mRCC patients treated with first-line sunitinib. Here, SII was associated with overall response rate (*p* < 0.0001) with median PFS of 6.3 vs. 18.7 months and median OS of 13.5 vs. 43.6 months in patients with SII ≥ 730 vs. SII < 730 (*p < 0*.0001, *p < 0*.0001, respectively) [43].

For patients treated with immune checkpoint monotherapy, several studies have explored the role of the neutrophil-to-lymphocyte and platelet-to-lymphocyte ratios in various types of cancers, including mRCC. A higher neutrophil-to-lymphocyte ratio and a higher platelet-to-lymphocyte ratio were associated with treatment failure and increased risk of death. However, lower neutrophil-to-lymphocyte ratio following treatment with nivolumab reportedly improved oncological outcomes, not taking into account the prognostic value of platelets [44,45,46]. In the prospective study by De Giorgi et al., with three hundred and thirteen mRCC patients treated with nivolumab in the Italian Expanded Access Programm, a high SII predicted worse OS in multivariate analysis (HR 2.99, 95%CI 2.09–4.31, *p < 0*.0001). Changes in SII at three months also predicted OS (*p < 0*.0001) [46]. To our knowledge, SII has been assessed only once in forty-three Japanese mRCC patients treated with first-line ipilimumab plus nivolumab. The one-year PFS rates were 90.0% and 54.8% for SII ≤561.7 and >561.7, respectively (*p* = 0.023). Unfortunately, no OS data have been published here to date [47]. The twelve-month PFS rates in our analysis were significantly lower at 34.6% for the overall population, with 45.9% for the SII low group, and 27.4% for the SII high group. However, the PFS rates shown in this analysis fit better with the data from the Checkmate 214 study, which showed an eighteen-month PFS rate of 43% [48].

There are some known confounders such as lifestyle, metabolic, and social factors that directly or indirectly affect the efficacy of IO therapies. Thus, patient physiology, inherent metabolic disorders such as obesity, smoking, sports, and alcohol consumption may affect the efficacy of IO therapies. Indeed, obesity and male sex were associated with the best IO effect [49]. In addition, with regard to drug interactions, no drug effects of widely used proton pump inhibitors were observed on the outcome of mRCC patients treated with ipilimumab plus nivolumab [50].

There are some limitations to this study. The relatively small sample size, the retrospective aspect, and short follow-up period is a drawback of this study. Further, we did not capture comorbidities such as autoimmune diseases, chronic medical conditions, body mass index (BMI), and smoking history at the time of SII measurement, which are important confounders given their strong effects on each variable. Therefore, prospective studies with large samples, broader collection of potential confounders, and longer follow-up periods are needed to confirm our results.

In summary, our findings suggest that SII is a prognostic and predictive marker in mRCC patients treated with first-line ipilimumab plus nivolumab and could help refine our clinical practice by helping us identify treatment strategies for mRCC patients.

## 5. Conclusions

This study highlights a high pre-therapy SII to be an independent predictive factor for poorer PFS and OS in mRCC patients treated with ipilimumab plus nivolumab in the first-line setting. Given the association of an SII increase during therapy with progression at initial imaging, SII may help distinguish between pseudo-progression and true progression on immune checkpoint inhibitor therapy. Thus, SII changes could be able to predict response and clinical outcome on ipilimumab plus nivolumab, providing a potentially simple tool to monitor treatment efficacy.

With the plethora of highly effective therapies now available, and consequently the need to balance costs and benefits, the development of a biomarker-based approach to treatment selection and therapy monitoring is essential. In this regard, a single biomarker such as a gene mutation or a gene expression signature appears not to be helpful due to the intra- and intertumorheterogeneity. Instead, it seems to be important to pursue an integrated biomarker approach using a composite biomarker that includes tumor characteristics, changes in the tumor microenvironment, and host factors like the microbiome. For mRCC, SII captures host and tumor factors, especially on the inflammatory level. It is easily and cost-effectively determined by routine laboratory testing, providing a broad availability.

## Figures and Tables

**Table 1:** Patients’ characteristics according to the peripheral blood SII level low (<788) vs. high (≥788) in patients with mRCC treated with first-line ipilimumab plus nivolumab.

	Overall, *n* = 49	Low SII (<788), *n* = 20	High SII (≥788), *n* = 29	*p*-Value
Age-median (IQR)				
RCC diagnosis	63.6 (39.9–82.7)	64.6 (46.9–79.9)	62.9 (39.9–82.7)	0.502
First metastasis	64.6 (39.9–83.5)	64.8 (46.9–81.8)	63.6 (39.9–83.5)	0.807
Male gender	35 (71.4%)	15 (75%)	20 (70%)	0.842
Karnofsky <80%	2 (4.1%)	0	2 (6.9%)	0.406
Histology				
Clear cell	39 (79.6%)	17 (85%)	22 (75.9%)	
Papillary	5 (10.2%)	1 (5%)	4 (13.8%)	
Chromophobe	1 (2.0%)	0	1 (3.5%)	
Other	3 (6.1%)	2 (10%)	1 (3.5%)	
NE	1 (2.0)		1 (3.5%)	0.578
pT stage				
pT1	8 (16.3%)	2 (10%)	6 (20.7%)	
pT2	5 (10.2%)	2 (10%)	3 (10.4%)	
pT3	24 (49.0%)	12 60%)	12 (41.4%)	
pT4	4 (8.2%)	0	4 (13.8%)	
NE	8 (16.3%)	4 (20%)	4 (13.8%)	0.361
*Nodal status*				
*pN0*	27 (55.1%)	11 (55%)	16 (55.2%)	
*pN+*	21 (42.9%)	8 (40%)	13 (44.8%)	
*pNx*	1 (2.0%)	1 (5%)	0	0.854
Metastasis				
synchronous	30 (61.2%)	13 (65%)	17 (58.6%)	
metachronous	19 (38.8%)	7 (35%)	12 (41.4%)	0.836
≥2 metastastic sites	36 (73.5%)	13 (65%)	23 (79.3%)	0.270
Prior curative metastasectomy	7 (14.3%)	4 (20%)	3 (10.3%)	0.347
Time from diagnosis to systemic treatment <1 year	37 (75.5%)	16 (80%)	21 (72.4%)	0.548
MSKCC score				
Good	4 (8.2%)	1 (5%)	3 (10.3%)	
Intermediate	39 (79.6%)	18 (90%)	21 (72.4%)	
Poor	5 (10.2%)	0	5 (17.2%)	
NE	1 (2.0%)	1 (5%)	0	0.538
IMDC score				
Good	2 (4.1%)	0	2 (6.9%)	
Intermediate	34 (69.4%)	16 (80%)	18 (62.1%)	
Poor	9 (18.4%)	1 (5%)	8 (27.6%)	
NE	4 (8.2%)	3 (15%)	1 (3.5%)	0.265
First imaging				
Progressive disease	23 (46.9%)	4 (20%)	19 (65.5%)	
Stable disease	7 (14.3%)	4 (20%)	3 (10.4%)	
partial response	18 (36.7%)	12 (60%)	6 (20.7%)	
NE	1 (2.0%)		1 (3.5%)	0.001 ***
Median time from primary tumor to metastasis (range, in months)	0 (0–198.4)	0 (0.33–118.5)	0 (0–198.4)	0.799
Median time to treatment (first diagnosis RCC to start ipilimumab plus nivolumab (range, in months)	4.41 (0.10–198.77)	3.93 (0.10–179.08)	5.10 (0.20–198.77)	0.669
Median time on ipilimumab plus nivolumab (range, in months)	4.64 (0.33–33.6)	6.64 (0.99–33.6)	3.42 (0.33–24.69)	0.014 *
Median follow up from start ipilimumab plus nivolumab to last follow up or death (range, in months)	9.53 (0.33–45.9)	17.4 (5.6–45.9)	7.36 (0.33–39.8)	0.008 **

Abbreviations: IMDC International Metastatic Renal Cell Carcinoma Database Consortium, IO immuno-oncology, IQR interquartile range, MSKCC Memorial Sloan-Kettering Cancer Center (Motzer) Score, PFS progression-free survival, Ref. reference, OS overall survival, SII systemic immune-inflammation index, * significant at 0.05 level, ** significant at 0.01 level, *** significant at 0.001 level.

**Table 2:** (**A**) Overview of calculated PFS depending on clinical parameters as well as SII score and univariate analyses of PFS, defined as time from start ipilimumab plus nivolumab to tumor progression. (**B**) Univariate analysis of OS, defined as time from start ipilimumab plus nivolumab to death/last follow-up, depending on clinical parameters and SII score.

(A)				
PFS				
Group under Investigation	Median (Months)	HR	95% CI	*p*-Value
SII Index < 788 (*n* = 20) vs. SII Index > 788 (*n* = 28)	9.04 (0.99–33.60) 4.01 (0.33–24.69)	1 2.70	0.22–5.97	0.014 *
SII Index < 854 (*n* = 22) vs. SII Index > 854 (*n* = 26)	8.29 (0.99–33.60) 4.18 (0.33–24.69)	1 2.15	1.01–4.55	0.046 *
Metastasis metachronous (*n* = 18) vs. synchronous (*n* = 30)	4.18 (0.33–24.69) 6.08 (0.66–33.60)	1 0.60	0.29–1.23	0.165
Time nephrectomy to metastasis ≥1 year (*n* = 12) vs. <1 year (*n* = 36)	4.64 (0.33–24.69) 5.29 (0.66–33.60)	1 0.82	0.37–1.79	0.615
MSKCC favorable (*n* = 4) vs. intermediate/poor (*n* = 43)	8.29 (2.63–24.69) 5.29 (0.33–33.60)	1 1.13	0.39–3.29	0.818
IMDC favorable (*n* = 2) vs. intermediate/poor (*n* = 43)	2.63 (2.63–16.27) 6.08 (0.33–33.60)	1 0.61	0.14–2.60	0.503
Index metastasis only one organ system (*n* = 12) vs. multiple organ systems (*n* = 36)	8.29 (2.53–33.60) 4.18 (0.33–30.94)	1 1.52	0.65–3.54	0.330
**(B)**				
**OS**				
**Group under Investigation**	**Median (Months)**	**HR**	**95% CI**	***p*** **-Value**
SII Index < 788 (*n* = 20) vs. SII Index > 788 (*n* = 29)	NR (5.59–45.90) 19.69 (0.33–39.81)	1 10.53	1.34–82.68	0.025 *
SII Index < 854 (*n* = 22) vs. SII Index > 854 (*n* = 27)	NR (3.58–45.90) 19.69 (0.33–39.81)	1 5.64	1.21–26.28	0.028 *
Metastasis metachronous (*n* = 18) vs. synchronous (*n* = 31)	NR (1.05–45.90) NR (0.33–39.81)	1 0.59	0.18–1.93	0.381
Time nephrectomy to metastasis ≥1 year (*n* = 12) vs. <1 year (*n* = 37)	NR (0.33–39.81) NR (1.05–45.90)	1 1.72	0.37–8.00	0.489
MSKCC favorable (*n* = 4) vs. intermediate/poor (*n* = 44)	NR (23.08–36.62) NR (0.33–45.90)	1 26.55	0.02–41,875.67	0.383
IMDC favorable (*n* = 2) vs. IMDC intermediate/poor (*n* = 43)	NR (23.09) NR (0.33–45.90)	1 23.34	0.00–55,5052.05	0.540
Index metastasis only one organ system (*n* = 13) vs. multiple organ systems (*n* = 36)	NR (4.64–39.81) NR (0.33–45.90)	1 3.92	0.50–30.67	0.193

Abbreviations: IMDC International Metastatic Renal Cell Carcinoma Database Consortium, PFS progression-free survival, OS overall survival, SII systemic immune-inflammation index, * significant at 0.05 level.

**Table 3:** Multivariate analysis of PFS (**A**) and OS (**B**) depending on clinical parameters and SII score. (**C**) Association of a change in SII (increase or decrease >20%) with treatment response at the time of initial imaging with ipilimumab plus nivolumab. An increase in SII of >20% above baseline at 12 weeks on therapy was significantly associated with tumor progression at initial imaging (chi-square test *p* = 0.003).

(A)				
PFS				
Group under Investigation	HR	95% CI	*p*-Value	
SII Index > 788 (ref. < 788)	3.63	1.35–9.74	0.010 **	
IMDC Intermediate/poor (ref. favorable)	2.31	0.33–16.16	0.400	
Index metastasis multiple organ systems (ref. only one organ system)	2.16	0.64–7.34	0.218	
Metastasis synchronous (ref. metachronous)	1.27	0.51–3.11	0.609	
**(B)**				
**OS**				
**Group under Investigation**	**HR**	**95% CI**	***p*** **-Value**	
SII Index > 788 (ref. < 788)	6.91	0.87–55.22	0.068	
IMDC Intermediate/poor (ref. favorable)	9.76	0.00-NR	0.977	
Index metastasis multiple organ systems (ref. only one organ system)	11.33	0.00-NR	0.974	
Metastasis synchronous (ref. metachronous)	2.64	0.74–9.44	0.135	
**(C)**				
**First Imaging**				
	**PR + SD**	**PD**	**Total**	***p*** **-Value**
**SII score on first imaging**				
**Increase >20%** **Decrease > 20% or stable**	4 21	13 10	17 31	
Total	25	23	48	
Pearson’s Chi-square (two-sided)				0.003 **
Fisher’s exact test (two-sided)				0.006 **

Abbreviations: IMDC International Metastatic Renal Cell Carcinoma Database Consortium, IO immuno-oncology, MSKCC Memorial Sloan-Kettering Cancer Center (Motzer) Score, PFS progression-free survival, PD progressive disease, PR partial response, Ref. reference, OS overall survival, SD stable disease, SII systemic immune-inflammation index, ** significant at 0.01 level.

## Data Availability

Not applicable.
